# Supplementary material for: Evolution of gene regulatory networks by means of selection and random genetic drift
Source: PeerJ. 2024 Aug 28;12:e17918. doi: 10.7717/peerj.17918 (PMC11365478; doi:10.7717/peerj.17918)
Supplement: Supplemental Information 1 [file peerj-12-17918-s001.pdf]

Evonet: Evolution of Gene Regulatory Networks

Authors:
Stefanos Papadantonakis, Antonios Kioukis, Charikleia Karageorkiou, Pavlos Pavlidis

About

Evonet is a stand-alone software simulating the evolution of GRNs under neutral and selective evolutionary pressure. Unlike previous implementations, the interactions between the genes take values from the whole set [-1,1], allowing for finer control and experiments.

Interplay between Recombination and Optimum state

We studied the effect of recombination on the time needed for the population to overcome fitness steps. We used EvoNET to simulate recombining and non-recombining datasets with given global optimum states. Then we subtracted the average time it took for each scenario to reach the specified average fitness value. Results are shown on Supplementary Figure S3. We report that different fitness optima show distinct behavior according to the presence of recombination. Namely fitness optima 1111111111 and 1111100000 seem to be traversed more quickly by recombining populations. Contrary, optima 1100110011 and 1010101010 display the opposite behavior. While these results are preliminary and could be the effect of initialization of the simulation, there seems to be evidence that suggest that the effect of recombination depends on the context of the network and should be explicitly studied.

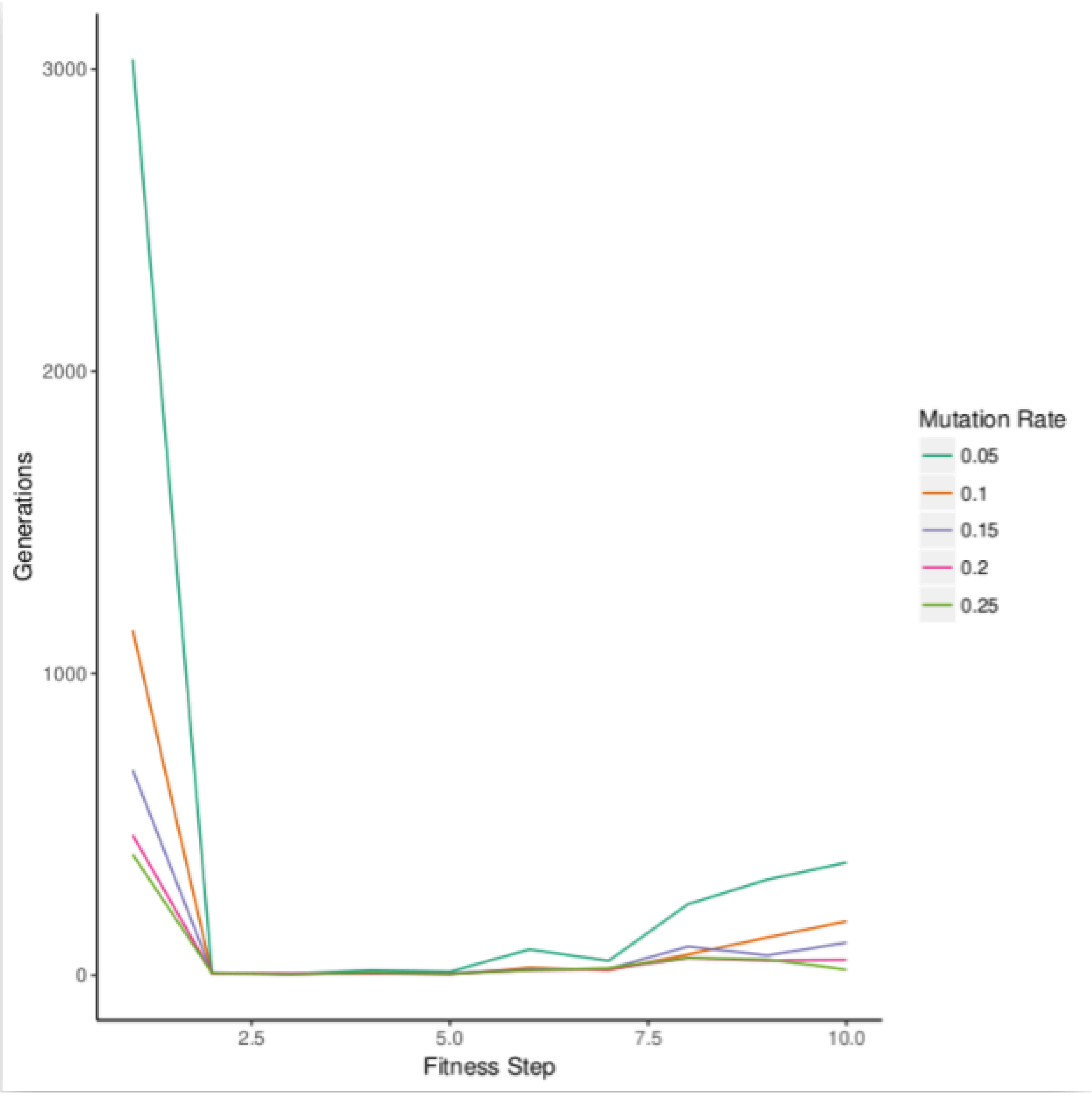

Supplementary Figure S1. Effect of mutation rate on the time between fitness steps. Increasing the mutation rate results in a reduction of the time needed to transition to the next step of the fitness landscape. 100 simulations were performed for each mutation rate value

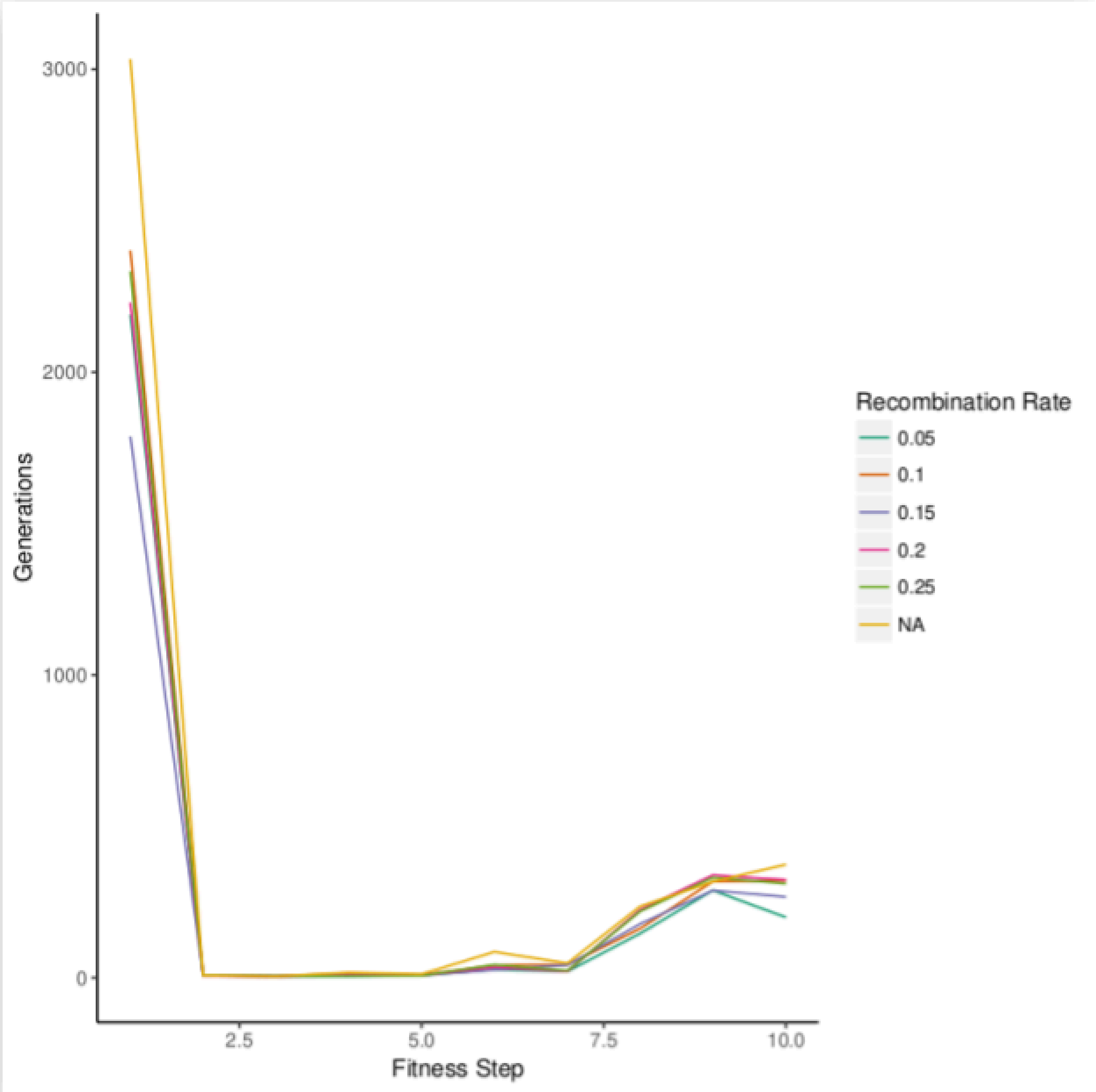

Supplementary Figure S2. The relationship between recombination rate and time needed to transition to the next step of the fitness landscape. We observed that intermediate values of recombination rates result in faster transition times, with the fastest times achieved with a rate of 0.15. Results were averaged over 100 simulations for each recombination rate.

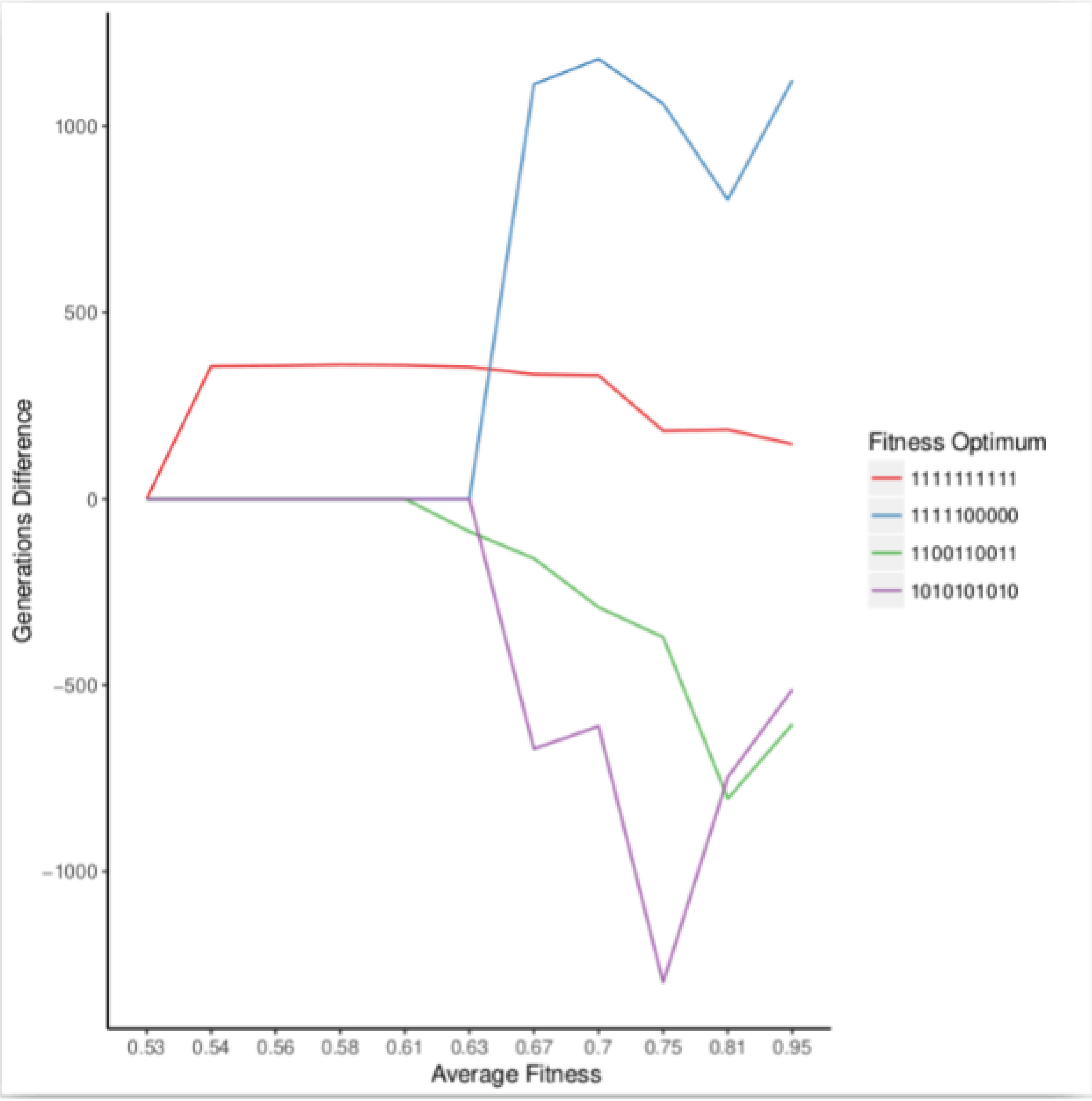

Supplementary Figure S3. Effect of presence of Recombination on the time of navigation of the fitness landscape according to the optimum state. We show the difference between recombining and non-recombining populations in time of reaching certain values of average fitness for different optimum vectors. Values greater than 0 mean that non recombining populations are slower to reach the specified fitness levels and vise versa. For each fitness optimum, 200 simulations were performed (100 incorporated recombination and 100 did not).

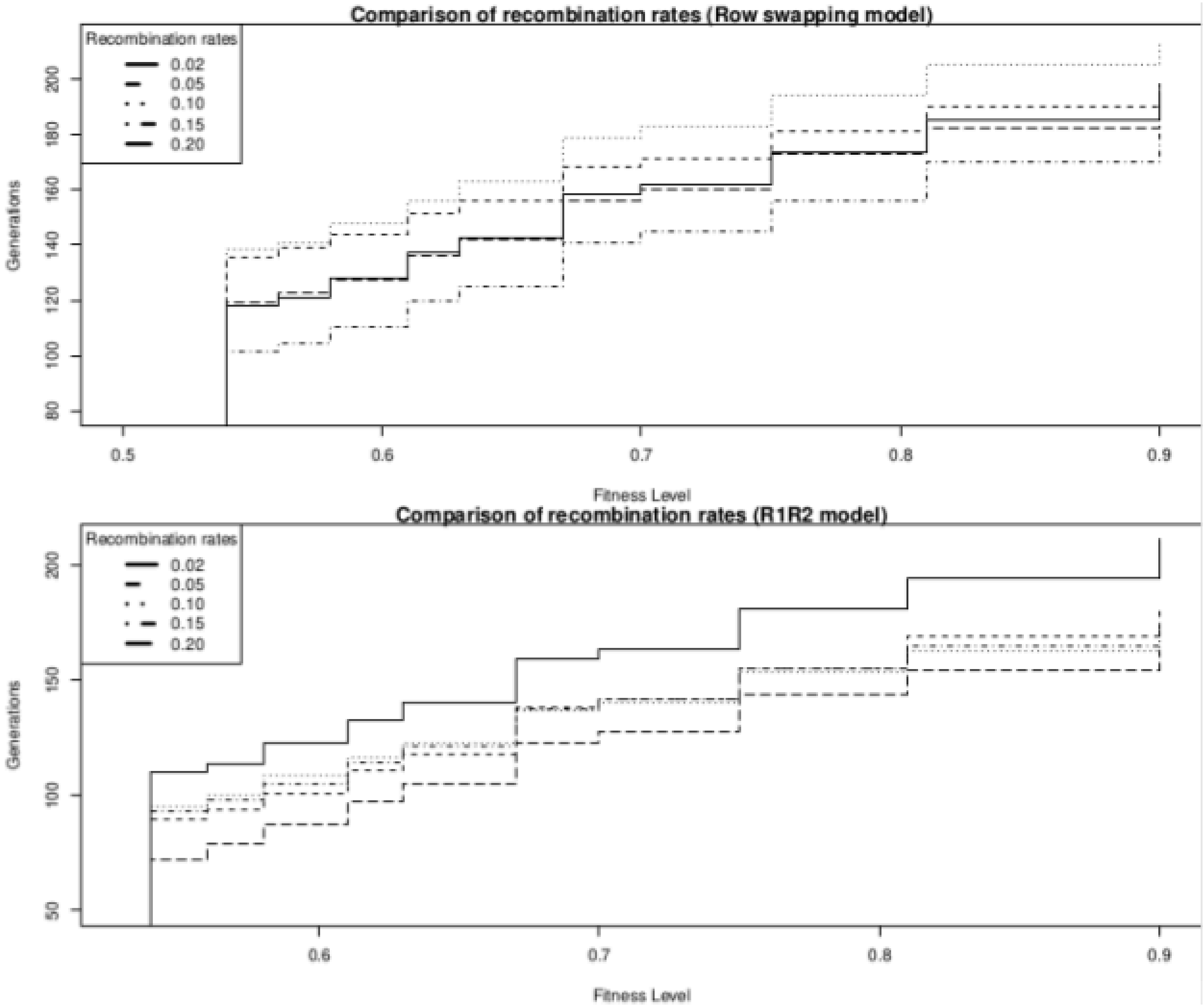

Supplementary Figure S4. Comparison between Wagner's swapping and the R1R2 recombination model regarding the number of generations required to traverse the fitness landscape. Results were averaged over 100 simulations for each recombination value.

Download and Compile

The following commands can be used to download the necessary prerequisite libraries before running Evonet:

$sudo apt-get install -y gsl-bin libgsl-dbg libgsl-dev

The above command is used to install the gsl library containing mathematical functions like binomial distribution.
To install the source code of the project run the following commands

$ mkdir evonet
$ cd evonet
$ wget https://github.com/antokioukis/evonet/archive/master.zip
$ unzip master.zip
$ cd evonet-master
$ make all

The executable is placed in the path evonet/evonet-master.
Test Run

To verify that evonet is installed correctly, a test run can be done with the following commands. These commands are going to create a small forward simulation and a GRN of 10 genes with a population size of 100.

$ ./evonet -N 100 -generations 100 -tarfit 0.5

If “Generation 0 Simulated.” is printed, evonet has been installed correctly.

In-tool Help

Evonet output a quick-reference help message that provides a short description for each of the supported command-line flags with the following command.

$ ./evonet –help

The quick reference help message generated by the current evonet release is the following:
-ploidy X: Number of parents per person (1 – 2)
-swapping X: R1R2_swapping or Row_swapping (Binary)
-min_R1R2 X: Minimum of R1R2 selection (Integer)
-max_R1R2 X: Maximum of R1R2 selection (Integer)
-min_count X: Minimum of gene counts (Integer)
-max_count X: Maximum of gene_counts selection (Integer)
-n X: Number of genes per person (Integer)

-mutrate X: Mutation Rate (Double)
-rob X: Check for robustness (Binary)
-num_of_rob_mutation X: Number of mutations per robustness check (Integer)
-rob_last On robust mutations change last bit of R1 R2 interactions (Binary)
-sense X: Number of sensitivity in R1 and R2 regions.(Integer)
-mod_change X: Number of generation after which the evolutionary model changes.
-recomb_rate X: Recombination Rate representing percentage(Float)
-seed X: Set the seed for the random distributions.
-tarfit X: Set the target average generation fitness of population.
-optimal_num X Set number of open genes instead of optimal vector.
-optimal_vec X: Set the target optimal which the population tries to reach.
-st_geno X: File where the starting genotypes can be read.
-N X: Number of persons to be simulated.(Integer)
-generations X: Number of generations to be simulated.(Integer)
-selection X: Inheritance based on fitness or neutral.(Binary)
-s2 X: s^2 for the fitness function.(Float)
-eN X Y : Create event at generation X, persons after event Y. (Integers)
-freq X: Frequency of export of data.(Integer)
-r1out X: Write R1_output at specified file.(File)
-r2out X: Write R2_output at specified file.(File)
-matout X: Write gene_interaction_matrix_output at specified file.(File)
-gencout X: Write gene_counts_output at specified file.(File)
-fitout X: Write fitness_output at specified file.(File)
-disout X: Write discrete_output at specified file.(File)
-robout X: Write robust_output at specified file.(File)
-gentpout X: Write genotype_occurrence_output at specified file.(File)

Input File Formats

Currently, evonet is able to produce all the necessary inputs that will later be required. If there are specific requirements use the -st_geno FILE option. FILE contains a space separated table containing the starting R1 and R2. The write_file.R which is contained in the previously downloaded files is used for easier creation of this optional input file.

Output Files

Evonet creates 9 files as output. Here we will use the default names, although each file's name can be chosen by the user.

R1.txt and **R2.txt** depict how the regulatory regions change as the forwarding continues. For parsing these files, you have to know the population size and the number of genes. Each discrete generation starts from line:

generation_numberpopulation_sizenumber_of_genes.

matrix.txt: contains the power of corresponding gene interaction of each individual in the population at a given generation. Each line of the file, should be interpreted row-wise in order to reconstruct the interaction matrix. For example if we have a simulation with 10 genes, and we want to find the interaction between the 3rd and the 4th gene we need the 23th element of the line.

counts.txt: contains the gene expression data produced by the simulation. It follows the same organizing scheme as R1.txt

discrete.txt: contains the discrete interaction data produced by the simulation. It follows the same organizing scheme as R1.txt

fitnesss.txt: contains data in 5 columns. The 1st shows the generation_number%2. The 2nd shows the population size, the 3rd depicts the number of the genes in the simulation. Finally, the 4th column shows the mean generation fitness and the last column the mutation rate used in this simulation.

robustness.txt: contains the gene interactions matrix after a robust generation was created. This is used to compare the contents of this file with the matrix.txt to see the level of similarity. It follows the organizing scheme of matrix.txt.

rob_discrete.txt: contains the discrete interaction data after a robust generation was created. This is used to compare the contents of this file with the discrete.txt to see the level of similarity. It follows the organizing scheme of discrete.txt.

genotype.txt: contains the number of different genotypes encountered at each generation as well as how prevalent is each genotype. So each line is a generation and if we have a line like 203 12 it means that we have 2 different genotypes and that the first is shared among 203 individuals and the other genotype is shared by 12 individuals.

infofile.txt: contains the command used for the simulation. This is useful for reproducibility purposes.

open_genes.txt: used when only a portion of the genes are under evolutionary pressure. It depicts how many genes that are evolving under neutrality are currently interacting with the GRN. It shares the organizing schemes of R1.txt except that for each individual only 1 number is outputted.

Analysis of the command-line parameters for the simulation:

Basic commands:

-ploidy: controls the ploidy of the population. Accepted input values 1,2. 1 specifies that each child of the next generation needs only 1 parent so no recombination event is possible. 2 specifies that a pair of parents is needed and allows the recombination models to take effect.

-swapping: specifies which recombination model is used. 0 is our model. 1 is the model proposed by Dr. Wagner.

-N: specifies the size of the initial population. Theoretically there is no limit of the population size but caution is advised due to non-linear relation between population size and running time.

-min_R1R2: controls the lowest level that R1 or R2 can be initialized. This is used when st_geno is unspecified.

-max_R1R2: controls the highest level that R1 or R2 can be initialized. This is used when st_geno is unspecified.

-min_count: controls the lowest level that gene expression counts can be initialized.

-max_count: controls the highest level that gene expression counts can be initialized.

-n: specifies how many genes are part of the GRN, regardless how many are under selective pressure.

-mutrate: specifies the mutation rate of the R1 and R2 regions. Used as argument in the poisson distribution describing the chance of mutation.

-generations: specifies how many generations will be created as part of the forwarding process.

-selection: for neutral evolution use 0 or selective pressure use 1.

-freq: control the frequency of data output.

-s2: specify how costly is a difference between each genotype and the optimum genotype.
Examples:
Create a run of neutral evolution on a population with 1000 population size,10 genes as part of the network, single parent inheritance, width of R1-R2 10-11, width of gene counts 10-11, mutation rate equal to 0.005, see the output of every 10th generation and we want 100 generations:

./evonet -ploidy 1 -N 1000 -min_R1R2 10 -max_R1R2 10 -min_count 10 -max_count 11 -mutrate 0.005 -n 10 -generations 100 -selection 0 -freq 10 -s2 5 -optimal_num 10

Based on the previous example, create a run with a specified selective pressure and also change the ploidy, and use the recombination model proposed by Dr. Wagner:

./evonet -ploidy 2 -swapping 1 -selection 1 -N 1000 -min_R1R2 10 -max_R1R2 10 -min_count 10 -max_count 11 -mutrate 0.5 -n 10 -generations 500 -freq 10 -s2 5 -optimal_num 10

Re-do the previous example, but now change the optimal fitness vector, the recombination model and stop when the population reaches a mean fitness of 0.91

```
./evonet -ploidy 2 -swapping 0 -selection 1 -tarfit 0.91 -N 1000 -min_R1R2 10 -max_R1R2 10
-min_count 10 -max_count 11 -mutrate 0.5 -n 10 -generations 10000 -freq 10 -s2 2
-optimal_vec 1110111101
```

On this example command-line, we provide a seed for the random time function instead of using the current time, and we provide the starting genotypes on R1R2_input.txt

```
./evonet -N 500 -st_geno R1R2_input.txt -seed 12345 -ploidy 2 -swapping 1 -selection 1 -tarfit 0.81
-min_count 10 -max_count 11 -mutrate 0.005 -n 10 -generations 100 -freq 10 -s2 5 -optimal_num 10
```

With the following command-line we specify that for every 10th generation we want to output the robustness data of the network. Each robust branch, will have additional 15 mutations and those mutations are free to change the last bit of the R1,R2 regions, that controls the type of interaction between 2 genes.

```
./evonet -num_of_rob_mutation 15, -rob_last 1 -rob 1 -ploidy 2 -swapping 0 -selection 1 -tarfit 0.91 -N
1000 -min_R1R2 10 -max_R1R2 10 -min_count 10 -max_count 11 -mutrate 0.005 -n 10 -generations 100
-freq 10 -s2 5 -optimal_num 10
```

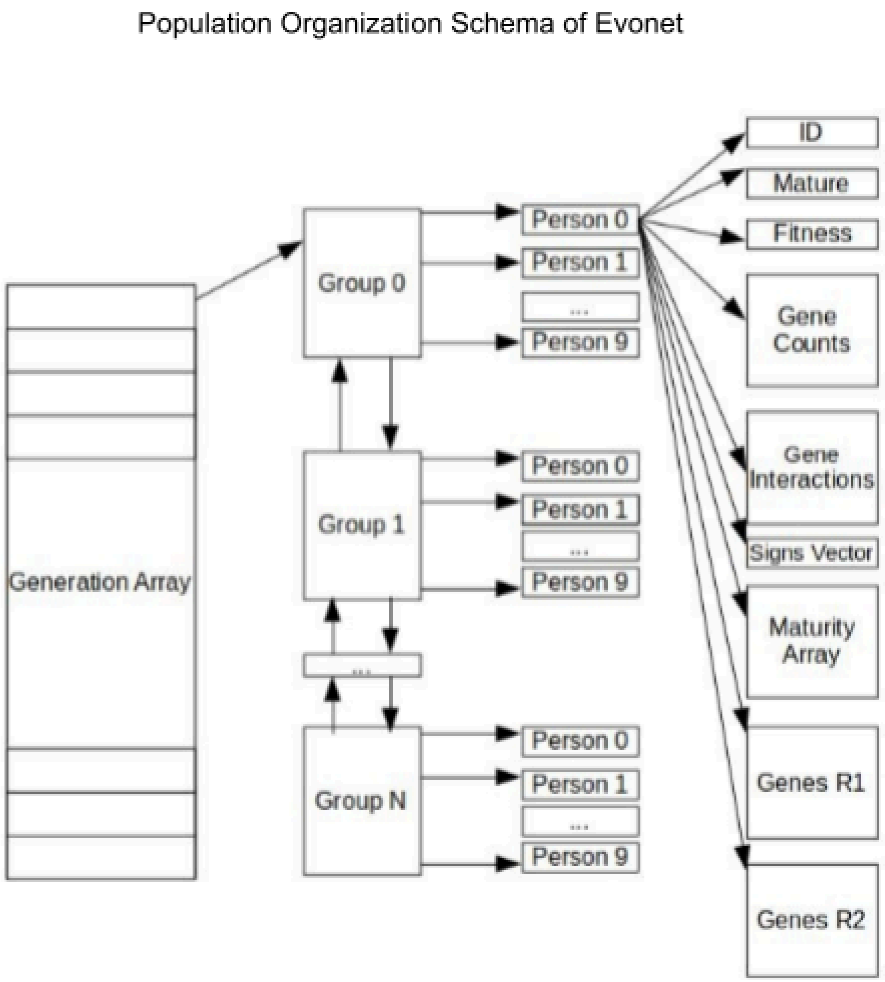

Supplementary Figure S5. Schematic representation of individuals in EvoNET.

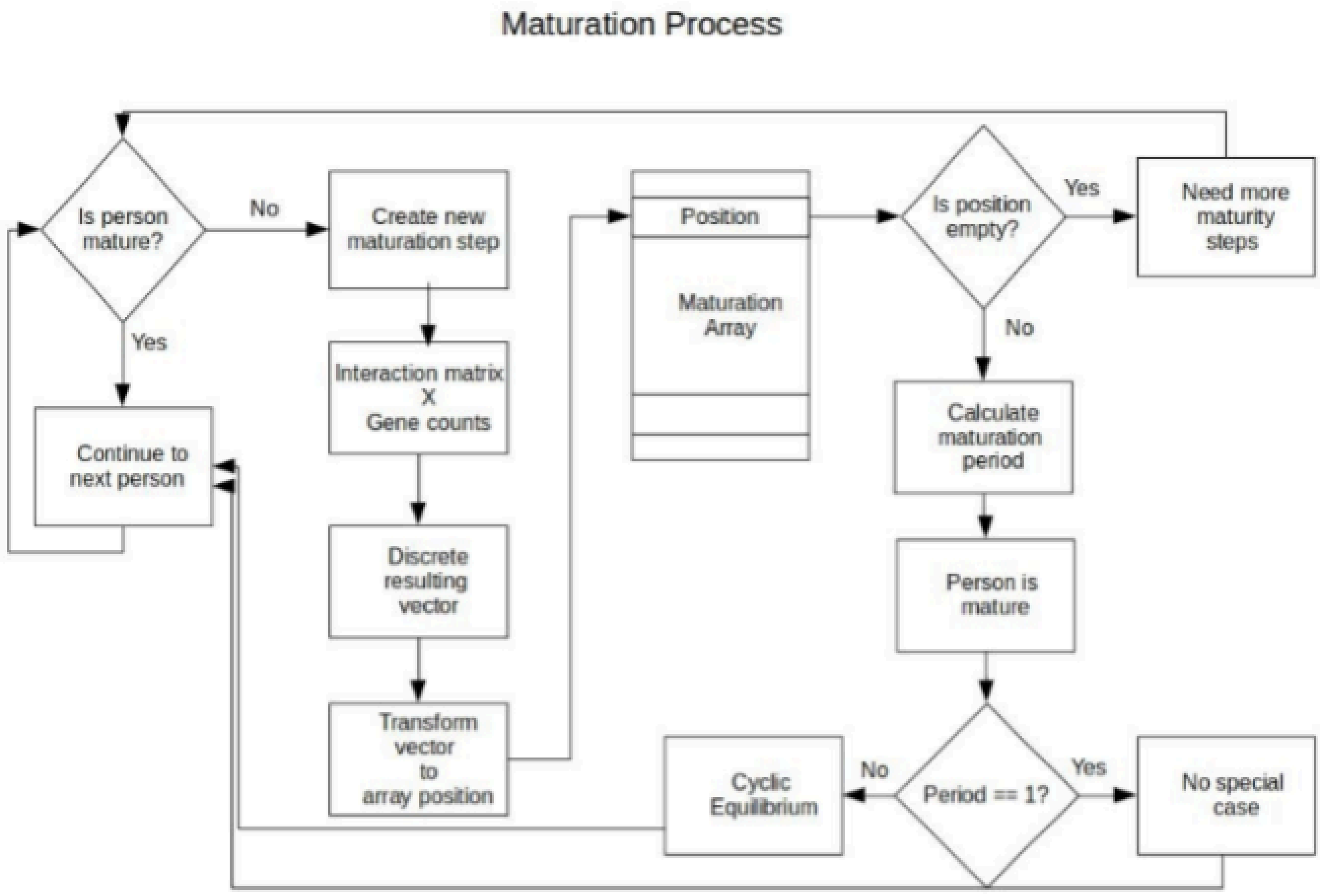

Supplementary Figure S6. Schematic representation of the maturation process in EvoNET.

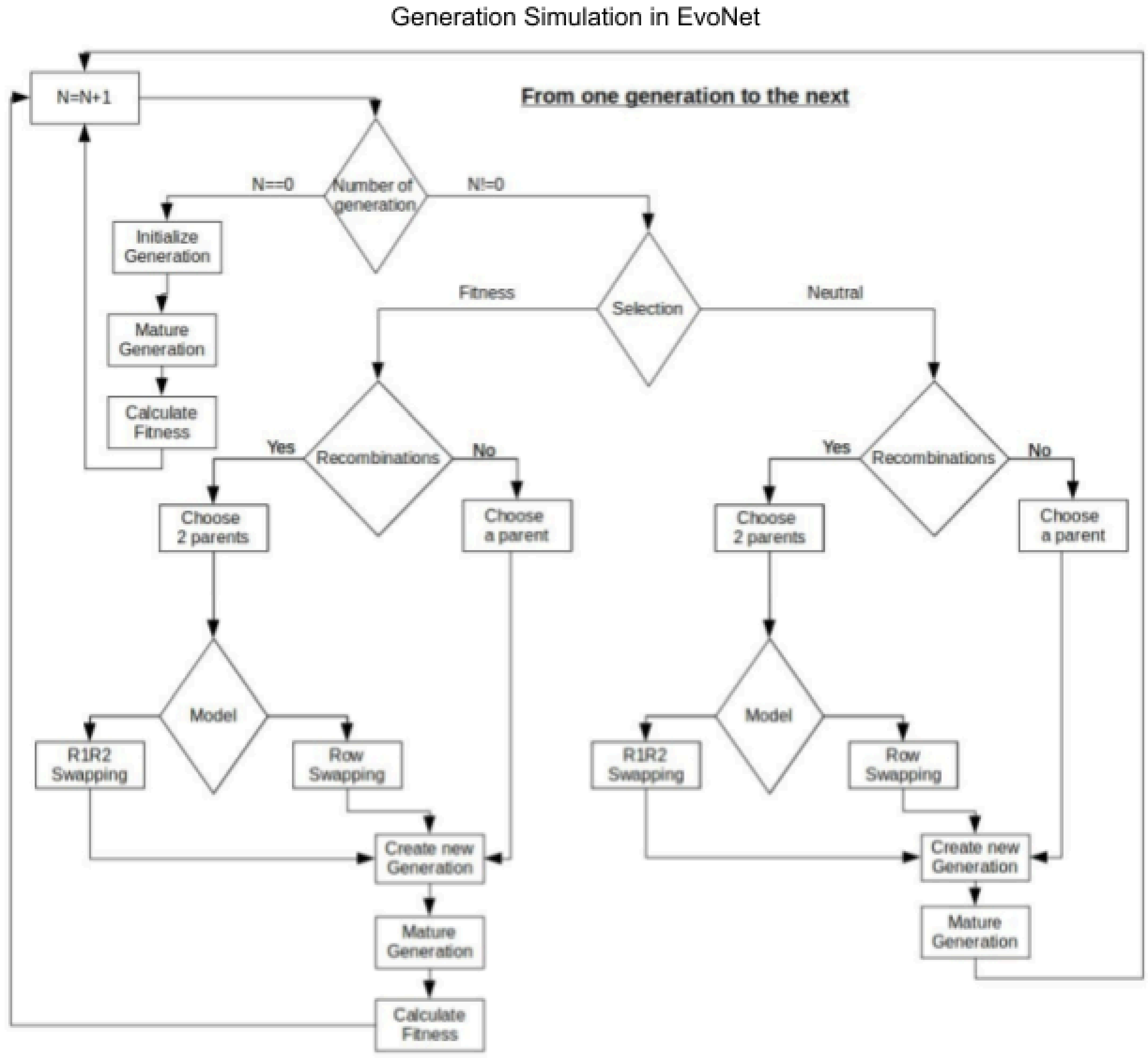

Supplementary Figure S7. Schematic representation of the processes within one generation in EvoNET.
